# New Spirotetronate Antibiotics, Lobophorins H and I, from a South China Sea-Derived *Streptomyces* sp. 12A35

**DOI:** 10.3390/md11103891

**Published:** 2013-10-15

**Authors:** Hua-Qi Pan, Song-Ya Zhang, Nan Wang, Zhan-Lin Li, Hui-Ming Hua, Jiang-Chun Hu, Shu-Jin Wang

**Affiliations:** 1 Department of Natural Products Chemistry, Shenyang Pharmaceutical University, Shenyang 110016, China; E-Mails: panhq@iae.ac.cn (H.-Q.P.); lzl1030@hotmail.com (Z.-L.L.); 2 Institute of Applied Ecology, Chinese Academy of Sciences, Shenyang 110016, China; E-Mails: zsykk123@163.com (S.-Y.Z.); wangn@iae.ac.cn (N.W.)

**Keywords:** lobophorins, spirotetronate antibiotics, antimicrobial, *Streptomyces*, South China Sea-derived actinomycete

## Abstract

Strain 12A35 was isolated from a deep-sea sediment collected from the South China Sea and showed promising antibacterial activities. It was identified as *Streptomyces* sp. by the 16S rDNA sequence analysis. Bioassay-guided fractionation using HP20 adsorption, flash chromatography over silica gel and octadecylsilyl (ODS) and semi-preparative HPLC, led to the isolation and purification of five metabolites from the fermentation culture of 12A35. Two new spirotetronate antibiotics, lobophorins H (**1**) and I (**2**), along with three known analogues, *O*-β-kijanosyl-(1→17)-kijanolide (**3**), lobophorins B (**4**) and F (**5**) were characterized by 1D, 2D-NMR and MS data. These compounds exhibited significant inhibitory activities against *Bacillus subtilis*. Compounds **1** and **5** exhibited moderate activities against *Staphylococcus aureus*. In particular, the new compound lobophorin H (**1**) showed similar antibacterial activities against *B. subtilis* CMCC63501 to ampicillin.

## 1. Introduction

Infectious disease is one of the most deadly diseases threatening public health. Meanwhile, drug-resistant bacteria are steadily rising, increasing the difficulty of treatment. Thus, an efficient way to address this issue is to develop new antibiotics both to help to overcome the antibiotic resistance and to treat new pathogenic diseases. Nowadays, natural products from marine-derived microorganisms have been an important source of novel lead structures for drug discovery. Marine actinomycetes are one of the most efficient groups of secondary metabolite producers. Many intriguing compounds with potent and various bioactivities have been found from marine microbes for lead compounds [1]. For instance, marinisporolides A and B were polyene macrolides isolated from the culture of an actinomycete from a new genus *Marinispora*. Marinisporolide A showed modest activity against *Candida albicans* [2]. The deep-sea-derived *Streptomyces* sp. SCSIO 03032 was capable of producing new bisindole alkaloids, spiroindimicins A–D, which exhibited moderate cytotoxicities against several cancer cell lines [3].

During our continuous screening for new antibiotics, the organic extract of the fermentation broth of a deep-sea-derived actinobacterial strain 12A35 showed potent antibacterial activities. Five spirotetronate antibiotics were isolated from an actinomycete 12A35 as a member of the genus *Streptomyces* by 16S rDNA analysis. Two compounds were determined to be new lobophorin analogues, designated as lobophorins H (**1**) and I (**2**), and the other three were characterized as known *O*-β-kijanosyl-(1→17)-kijanolide (**3**), lobophorins B (**4**) and F (**5**). Notably, the new compound lobophorin H (**1**) showed similar antibacterial activities against *Bacillus subtilis* to ampicillin as a positive control drug.

## 2. Results and Discussion

### 2.1. Taxonomy of the Strain 12A35

**Figure 1:**
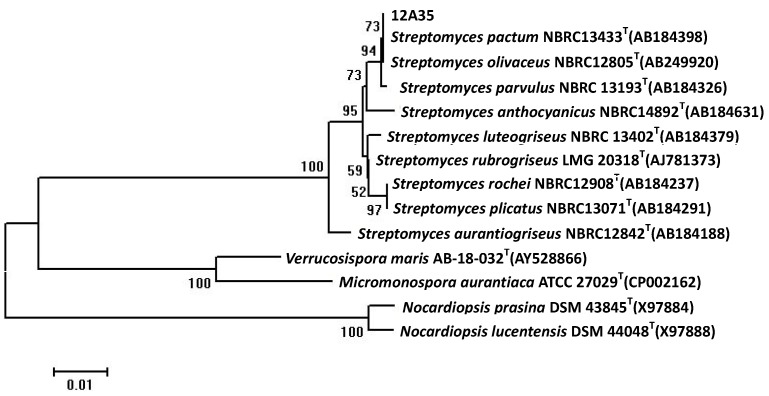
Phylogenetic tree of 16S rDNA sequences of 12A35 strain by the neighbor-joining method.

The 16S rDNA of producing strain 12A35 was polymerase chain reaction (PCR) amplified and sequenced. The strain 12A35 16S ribosomal RNA gene was submitted in the GenBank Database with the accession number KF313921. Sequence analysis showed that 16S rRNA gene sequence of 12A35 to be most similar to those of *Streptomyces pactum* NBRC 13433^T^, *Streptomyces olivaceus* NBRC 12805^T^, and *Streptomyces parvulus* NBRC 13193^T^, with sequence identities of 100%, 100%, and 99.33%, respectively. The phylogenetic tree generated by a neighbor-joining method based on 16S rRNA gene sequence clearly revealed the evolutionary relationship of the strain 12A35 with a group of *Streptomyces* species (Figure 1). So, this strain was designated as *Streptomyces* sp. 12A35.

### 2.2. Structure Determination

**Figure 2:**
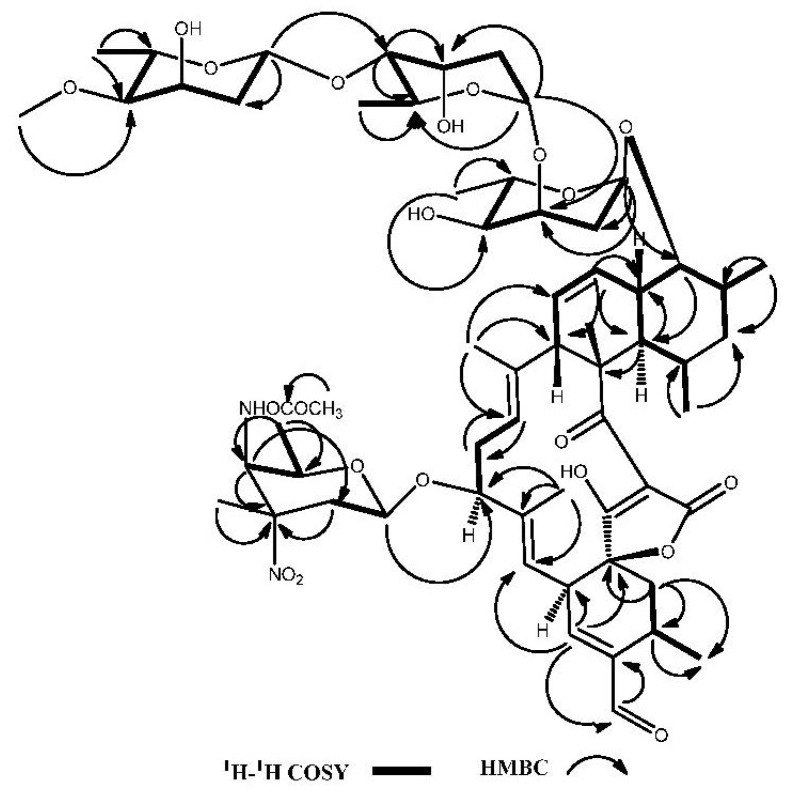
Selected key ^1^H-^1^H COSY and HMBC correlations of lobophorin H (**1**).

**Figure 3:**
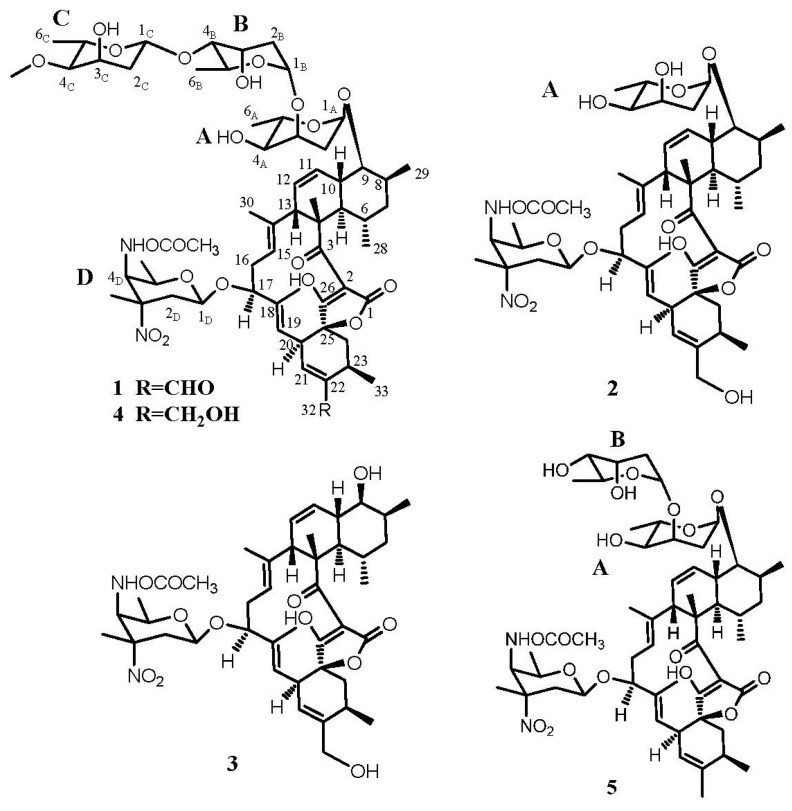
Chemical structures of compounds **1**–**5**.

Compound **1** was obtained as a white powder. Its high-resolution electrospray ionization mass spectrometry (HRESIMS) exhibited a [M − H]^−^ ion at *m*/*z* 1183.5807, corresponding to a molecular formula of C_61_H_88_N_2_O_21_, with 19 degrees of unsaturation. The ^1^H NMR (600 MHz, CDCl_3_) spectrum (Table 1) exhibited four methyl singlets at δ 1.35, 1.45, 1.61, 1.59 and seven methyl doublets at δ 0.64, 1.09, 1.16, 1.20, 1.25, 1.26, 1.36, two methoxy groups at δ 3.41 and 3.72, five olefinic protons at δ 5.17, 5.28, 5.38, 5.73, 6.56 and one aldehyde at δ 9.51. The ^13^C NMR (150 MHz, CDCl_3_) (Table 1) and HSQC spectra revealed 61 carbon signals, including four carbonyls (including an aldehyde), ten olefinic carbons, four sugar anomeric carbons, eleven methyls. The ^1^H and ^13^C NMR spectra readily indicated the presence of four 2,6-dideoxysugar units through easily identifiable signals for anomeric and 6-methyl protons and carbons. The four monosaccharide moieties were identified as two digitoxose, one 4-*O*-methyl-digitoxose and one kijanose by the interpretation of ^1^H-^1^H COSY, HMQC and HMBC spectra. The remaining 33 signals were suggestive of a spiroteronate skeleton [4,5]. Further, compound **1** showed similar ^1^H and ^13^C NMR spectra to lobophorin B (**4**) except for the hydroxymethyl signals at C-22 [4]. The oxygenated methylene (δ_H_ 4.22 (2H, m, H-32) and δ_C_ 64.9 (C-32)) in lobophorin B was replaced by an aldehyde group at δ_H_ 9.51 (1H, s, H-32) and δ_C_ 193.3 (C-32) in **1**, which resulted downfield shift of H-21 proton signal from δ 5.50 to δ 6.55. The HMBC correlations (Figure 2) from proton signals at δ_H_ 9.51 (1H, s, H-32) to δ_C_ 144.6 (C-22) and 25.2 (C-23), as well as from δ_H_ 6.55 (1H, s, H-21) to δ_C_ 193.3 (C-32), confirmed the aldehyde group located at C-22 in compound **1**, designated as lobophorin H (Figure 3). Furthermore, the similar coupling constant values of **1** to lobophorin B indicated the same relative stereochemistry [5]. The relative configuration of anomeric carbon of the sugars was easily determined to be α-configuration for sugars A and B and β-configuration for sugars C and D from the coupling constants for anomeric protons. The NOESY correlations between H-5/H-9, H-8/H-9, H-10/H-6, H-10/H-29, H-13/H-15 and H-16/H-30 supported the above relative stereochemistry.

**Table 1:** ^1^H and ^13^C NMR data of compounds **1**–**4** in CDCl_3_.

Position	1		2		3	4	Lobophorin F [6]
	^δ^C	^δ^H ( *J* in Hz)	^δ^C	^δ^H ( *J* in Hz)	^δ^C	^δ^C	^δ^C
1	166.71		167.24		167.12	167.23	167.3
2	101.68		101.85		101.89	101.76	101. 9
3	206.47		206.07		206.43	206.33	206.3
4	50.93		50.90		51.02	50.92	51.0
5	43.11	2.00 (m)	43.20	1.99 (m)	42.79	43.10	43.4
6	31.28	1.61 (m)	31.29	1.61 (m)	31.19	31.30	31.2
7	41.66	1.58 (m), 1.50 (m)	41.73	1.59 (m), 1.51 (m)	41.72	41.69	41.9
8	34.37	2.22 (m)	34.98	1.89 (m)	34.71	34.39	34.5
9	84.08	3.44 (m)	85.57	3.55 (dd, 10.9, 5.4)	76.08	84.16	86.7
10	38.39	2.08 (m)	38.37	2.15 (m)	39.22	38.42	38.2
11	125.97	5.73 (d, 10.6)	124.98	5.71 (d, 10.9)	125.51	125.84	125.9
12	126.29	5.38 (m)	127.18	5.38 (d, 9.7)	126.51	126.45	126.6
13	53.24	3.46 (m)	52.98	3.48 (m)	53.17	53.15	53.1
14	135.92		135.45		135.83	135.73	135.7
15	123.22	5.17 (d, 9.2)	123.59	5.16 (d, 9.3)	123.32	123.34	123.5
16	30.02	2.34 (m), 1.71 (m)	30.94	2.34 (m), 2.25 (m)	31.08	30.03	31.2
17	78.69	4.22 (m)	78.33	4.19 (m)	78.47	78.36	78.9
18	139.46		137.09		137.03	137.08	136.7
19	116.72	5.28 (d, 10.5)	119.17	5.11 (d, 10.9)	119.23	119.19	119.9
20	41.24	3.80 (d, 11.1)	40.17	3.85 (d, 10.9)	40.18	40.17	40.4
21	148.12	6.56 (s)	121.52	5.49 (s)	121.43	121.52	120.6
22	144.59		141.24		141.35	141.28	137.8
23	25.36	3.01 (m)	27.87	2.66 (m)	27.93	27.95	31.9
24	34.80	2.36 (m), 1.89 (m)	35.31	2.37 (m), 1.82 (m)	35.33	35.35	35.5
25	82.94		83.32		83.24	83.30	83.3
26	200.97		201.37		201.49	201.63	201.7
27 (4-CH_3_)	15.13	1.62 (s, 3H)	14.98	1.59 (s, 3H)	15.03	15.05	15.1
28 (6-CH_3_)	22.23	0.64 (d, 4.9)	22.15	0.63 (d, 5.8)	22.28	22.23	22.2
29 (8-CH_3_)	14.11	1.09 (d, 6.4)	14.39	1.09 (d, 7.0)	12.99	14.12	14.6
30 (14-CH_3_)	13.73	1.35 (s, 3H)	13.70	1.31 (s, 3H)	13.70	13.70	13.8
31 (18-CH_3_)	15.18	1.45 (s, 3H)	15.03	1.45 (s, 3H)	15.11	15.09	15.1
32 (22-C)	193.32	9.51 (s)	64.85	4.20 (s, 2H)	64.93	64.89	21.8
33 (23-CH_3_)	19.97	1.36 (d, 6.4, 3H)	20.14	1.30 (m, 3H)	20.18	20.18	20.2
A1	98.00	4.78 (d, 4.7)	99.58	4.90 (d, 2.7)		97.98	99.1
A2	31.02	2.30 (m), 2.35 (m)	34.05	2.25 (m, 2H)		30.95	33.4
A3	66.61	4.00 (m)	67.26	3.98 (m)		66.67	74.1
A4	71.80	3.26 (dd, 8.7, 3.5)	72.60	3.19 (d, 8.9)		71.81	72.4
A5	64.94	4.00 (m)	65.23	3.82 (m)		64.95	65.0
A6	17.72	1.26 (d, 6.0, 3H)	17.69	1.30 (m, 3H)		17.71	17.7
B1	90.97	5.13 (m)				90.99	96.5
B2	34.08	2.12 (m), 1.91 (m)				34.07	35.3
B3	65.48	4.22 (m)				65.53	66.9
B4	82.10	3.23 (dd, 7.7, 2.3)				82.1	72.5
B5	62.12	3.98 (m)				62.13	65.6
B6	17.89	1.20 (d, 6.0, 3H)				17.87	17.7
C1	98.28	4.91 (dd, 9.3, 2.0)				98.30	
C2	36.65	2.16 (m), 1.67 (m)				36.64	
C3	63.96	4.25 (d, 2.6)				63.96	
C4	82.10	2.84 (dd, 9.2, 2.6)				82.05	
C5	68.37	3.76 (q, 5.8)				68.37	
C6	18.25	1.25 (d, 6.0, 3H)				18.25	
C4-OCH_3_	57.37	3.41 (s, 3H)				57.37	
D1	97.39	4.46 (dd, 9.2, 1.5)	96.98	4.42 (dd, 9.8, 1.9)	97.03	96.96	97.4
D2	35.72	2.77 (m), 1.63 (m)	35.65	2.75 (m), 1.59 (m)	35.71	35.71	35.8
D3	91.09		90.98		91.01	91.12	90.9
D4	53.72	4.39 (d, 10.4)	53.66	4.35 (d, 10.1)	53.66	53.67	53.9
D5	69.12	3.48 (m)	69.07	3.47 (d, 7.4)	69.07	69.05	68.9
D6	16.97	1.16 (d, 6.0, 3H)	16.94	1.16 (d, 6.4, 3H)	16.97	16.97	17.0
D3-CH_3_	25.22	1.59 (s, 3H)	25.25	1.56 (s, 3H)	25.28	25.27	25.3
D4 C=O	157.34		157.44		157.35	157.38	157.4
D4-OCH_3_	52.74	3.72 (s, 3H)	52.72	3.72 (s, 3H)	52.69	52.73	52.7

Compound **2** was obtained as a white amorphous powder. The HRESIMS data (*m*/*z* 911.4544 [M − H]^−^ and *m*/*z* 913.4522 [M + H]^+^) of **2** established the molecular formula to be C_48_H_68_N_2_O_15_. The ^1^H NMR (600 MHz, CDCl_3_) spectrum (Table 1) showed four methyl singlets at δ 1.31, 1.45, 1.56, 1.59 and five methyl doublets at δ 0.63, 1.09, 1.16, 1.30, 1.30, a methoxy groups at δ 3.72, one oxygenated methylene at δ 4.20 and five olefinic protons at δ 5.11, 5.16, 5.38, 5.49, 5.71. The ^13^C NMR (150 MHz, CDCl_3_) (Table 1) and HSQC spectra revealed 48 carbon signals, including three carbonyls, ten olefinic carbons, two sugar anomeric carbons, nine methyls. According to the ^1^H and ^13^C NMR spectral data, compound **2** was determined as an analogue of compound **1**. Comparing with the ^1^H and ^13^C NMR data of lobophorin B (**4**), the signals of sugar B (digitoxose) and sugar C (4-*O*-methyl-digitoxose) in lobophorin B were absent in compound **2** (Table 1). The HMBC correlations between δ_H_ 4.79 (H-1A), 2.25 (H-2A) and δ_C_ 67.26 assigned the carbon signal at δ 67.26 to C-3A. In comparison with ^13^C-NMR data of lobophorin F, 6.8 ppm upfield deglycosidation shift of in C-3A was consistent with the loss of the sugar B and C moieties in **2**. The ^1^H- and ^13^C-NMR signals of **2** were assigned by HSQC, HMBC and ^1^H-^1^H COSY spectral analyses (Table 1). Based on these MS and NMR data, the structure of **2** was determined to be lobophorin I (Figure 3). Furthermore, the relative stereochemistry could be determined by comparison of NMR data with **1**.

Compound **3** was isolated as a white powder. Its molecular formula was determined as C_42_H_5__8_N_2_O_12_ by HRESIMS (*m*/*z* 781.3917 [M − H]^−^). By comparing ^1^H NMR (600 MHz, CDCl_3_) and ^13^C NMR (150 MHz, CDCl_3_) data (Table 1) with those previously reported, **3** was identified as *O*-β-kijanosyl-(1→17)-kijanolide (Figure 3) which was reported as a methanolysis product of kijanimicin [7]. Compound **3** was found for the first time as a natural product.

Compound **4**, white powder, afforded HRESIMS data (*m*/*z* 1185.5970 [M − H]^−^ and 1209.5901 [M + Na]^+^) consistent with the molecular formula C_61_H_90_N_2_O_21_. According to ^1^H NMR (600 MHz, CDCl_3_) and ^13^C NMR (150 MHz, CDCl_3_) (Table 1), **4** was identified as lobophorin B (Figure 3), which was previously found from the fermentation broths of a marine bacterium showed potent antiinflammatory activities in the PMA (Phorbol-Myristate-Acetate)-induced mouse ear edema model [5].

Compound **5** was obtained as a white powder, whose HRESIMS gave the molecular formula C_54_H_7__8_N_2_O_17_ base on quasi-molecular ion peaks at *m*/*z* 1049.5055 [M + Na]^+^ and 1025.5203 [M − H]^−^. The ^1^H NMR (600 MHz, CDCl_3_) data of **5** was identical to those published data of lobophorin F (Figure 3), which was previously isolated from a *Streptomyces* sp. SCSIO 01127 and showed antibacterial activities and cytotoxic activities [6].

The spirotetronate antibiotics are a class of natural products that exhibit broad biological activities, including antibacterial, antitumor, antiviral, antimalaria effects and cholesterol biosynthesis inhibition [5,8,9]. They feature an unusual macrolide that contains a characteristic tetronic acid (spiro-linked to a cyclohexene ring) conjugated with a trans-decalin system either by a carboxylic ester or a carbonyl group [10]. In order to continue to search for new drugs, these compounds are continually found including kijanimicin [11], tetrocarcins [12], chlorothricin [10], decatromicins [13], saccharocarcins [14], antlermicins [15], versipelostatins [16], arisostatins [17], quartromicins [9] and chrolactomycin [18]. One representative of this group is lobophorins, among which lobophorins A and B were produced from an alga-associated acintobacterium [5], lobophorins C and D were produced by a marine spongerelated *Streptomyces* [19], and lobophorins E, F and G were isolated from marine-derived *Streptomyces* [6,20]. The discovery of more spirotetronate antibiotics will throw more light on the structure-activity relationships and potential applications of these compounds.

### 2.3. Antimicrobial Activity of Compounds 1–5

Regarding the results shown in Table 2, the tested lobophorins did not exhibit inhibitory activity on Gram-negative bacteria (*E. coli*) and fungi (*C. albicans*, *F. moniliforme*). Only compounds **1** and **5** exhibited moderate activities against *Staphylococcus aureus* ATCC29213 with minimum inhibitory concentrations (MIC) values of 50 and 6.25 μg·mL^−1^, respectively. All the tested compounds exhibited inhibitory activities against *Bacillus subtilis* CMCC63501. Compounds **1** and **4** showed strong activities against *Bacillus subtilis* CMCC63501 with MIC values of 3.13 and 1.57 μg·mL^−^^1^, respectively, while compounds **2**, **3** and **5** possessed moderate activities against *Bacillus subtilis* CMCC63501 with MIC values of 6.25, 50, 50 μg·mL^−^^1^, respectively. With the increase of the amount of monosaccharide units, the inhibitory activity increased indicating that monosaccharide might play an important role for the antimicrobial activity of lobophorins. These findings support the proposal that the change of the length of saccharide chains alters the biological activity of the natural product, in which the sugars contribute to specific interactions with the biological target [21]. It is noticeable that lobophorin H (**1**) showed similar antibacterial activities against *Bacillus subtilis* CMCC63501 to ampicillin. Given the significant inhibitory activities against Gram-positive bacteria, lobophorins F (**5**) and H (**1**) may potentially find applications in anti-infective drug development.

**Table 2:** Minimum inhibitory concentrations for compounds **1**–**5** (μg·mL^−1^).

Compounds	*S. aureus* ATCC29213	*B. subtilis* CMCC63501	*E. coil* ATCC25922	*C. albicans* ATCC10231	*F. moniliforme* S16
**1**	50	1.57	>100	>200	>200
**2**	>100	50	>100	>200	>200
**3**	100	50	>100	>200	>200
**4**	100	3.13	>100	>200	>200
**5**	6.25	6.25	>100	>200	>200
ampicillin	3.13	1.57	25	NA	NA
nystatin	NA	NA	NA	6.25	25

NA: Not assayed.

## 3. Experimental Section

### 3.1. General Experimental Procedures

The chromatographic silica gel (500–600 mesh) was purchased from Qingdao Ocean Chemical Factory (Qingdao, China) and Diaion HP 20 macroporous resin was purchased from Mitsubishi Chemical Co., Ltd., Tokyo, Japan. RP-HPLC analysis and semi-preparation were conducted using a U3000 HPLC system (Dionex, Sunnyvale, CA, USA) and performed with a C18 YMC-Pack ODS-A column (5 μm, φ 10 × 250 mm). HRESIMS was recorded on a Bruker QTOF-ESI mass spectrometer. One-dimensional and two-dimensional NMR spectroscopy (^1^H-NMR, ^13^C-NMR, HSQC, HMBC, ^1^H-^1^H COSY) were conducted with a Bruker AV600 spectrometer (Rheinstetten, Germany). Deuterated NMR solvents were purchased from Cambridge Isotopes (Andover, MA, USA). Specimens of the strains for antimicrobial activity were deposited at the Group of Microbial Biotechnology, Institute of Applied Ecology, Chinese Academy of Sciences.

### 3.2. Microorganisms and Fermentation Conditions

The sediment was collected from the South China Sea at the depth of 2134 m (17°59.928′N, 111°36.160′E). To effective isolation of culturable marine actinomycetes by making the culture conditions similar to true marine environment, the strain 12A35 was isolated after incubation at 28 °C for 1 week on modified Gauze’s synthetic medium NO. 1 with artificial sea water instead of NaCl and distilled water (soluble starch 20.0 g; KNO_3_ 1.0 g; MgSO_4_·7H_2_O 0.5 g; K_2_HPO_4_ 0.5 g; FeSO_4_·7H_2_O 10.0 mg; agar 15.0 g; artificial sea water 1.0 L; adjust pH 7.0). Genomic DNA isolation, PCR amplification of 16S rDNA, and sequencing were performed with conventional methods. Sequence analysis of 16S rDNA were performed using BLASTN. Phylogenetic tree were constructed using Neighbor-Joining method of MEGA (version 5.0). Tree topologies were evaluated by bootstrap analysis with 1000 replicates. 

The strain 12A35 was maintained on modified Gauze’s synthetic medium NO. 1 at 28 °C, and the agar was cut into pieces (1 × 1 cm) and inoculated into 5 × 100 mL of seed medium (potato sucrose broth containing sucrose 20 g, infusion from 200 g potatoes, artificial sea water 1 L at pH 6.0 before sterilization) in 500-mL Erlenmeyer flasks, then cultivated at 28 °C for 2 days with shaking at 180 rpm. Seed cultures were transferred into 24 × 650 mL production medium in 3.0 L Erlenmeyer flasks, with an inoculation volume of 3%–4% (v/v), and incubated under the same conditions for 7 days.

### 3.3. Bioactivity-Guided Isolation and Purification

Fermentation broth (16 L) of 12A35 was centrifuged at 4000× *g* for 30 min. The supernatant was subjected to HP20 macroporous adsorption resin by two-step gradient elution with EtOH/H_2_O solutions of 10% and 100%. The EtOH fraction was evaporated to dryness under vacuum with a rotary evaporator. The mycelia were extracted three times with 3 L acetone and the acetone solution was evaporated to dryness. Two above residues were combined and extracted three times with methanol as the crude extract for further isolation. The crude extract (30.04 g) were subjected to vacuum flash chromatography over silica gel (500–600 mesh) and gradiently eluted with dichloromethane and methanol at ratios of (v/v) 100:0, 98:2, 95:5, 90:10, 80:20, 70:30, 60:40, 50:50 and 0:100 to give nine fractions (f1–f9). F4 (yield: 1.77 g) obtained by elution with dichloromethane/methanol (90:10, v/v) showed antibacterial activity. F4 was subjected to vacuum flash chromatography over ODS and eluted with CH_3_OH/H_2_O (1:9–10:0), to give ten sub-fractions (rf1–rf10). Sub-fractions rf8 (458 mg) and rf9 (698 mg) with antibacterial activities were combined, and further separated by a semi-preparative HPLC system (Dionex U3000, Sunnyvale, CA, USA) using a C18 YMC-Pack ODS-A column (5 μm, φ 10 × 250 mm) eluted with 90% methanol containing 0.05% trifluoroacetic acid (TFA) at a flow rate of 2.5 mL/min with UV detection at 220 nm. Compounds **1** (18.1 mg), **2** (12.4 mg), **3** (6.3 mg), **4** (67.3 mg), and **5** (11.6 mg) were obtained at retention time of 15.2 min, 9.6 min, 8.9 min, 11.5 min, 16.8 min, respectively.

### 3.4. Antimicrobial Activity of Compounds 1–5

Antimicrobial activity of compounds **1**–**5** were performed as described methods previously [22], and the minimum inhibitory concentrations (MIC) of compounds (**1**–**5**) were determined against five microbial strains, including *Staphylococcus aureus* ATCC 29213, *Bacillus subtilis* CMCC63501, *Escherichia coil* ATCC25922, *Candida albicans* ATCC10231 and *Fusarium moniliforme* S16. Ampicillin for bacteria and nystatin for fungi were used as positive controls with medium as a negative control. 

## 4. Conclusions

Two new spirotetronate antibiotics lobophorins H (**1**) and I (**2**), along with three known analogues, *O-*β-kijanosyl-(1→17)-kijanolide (**3**), lobophorins B (**4**) and F (**5**) were isolated and characterized from a deep-sea-derived *Streptomyces* sp. 12A35. These compounds exhibited significant inhibitory activities against *Bacillus subtilis*. Compounds **1** and **5** exhibited moderate activities against *Staphylococcus aureus*. Notablely, the compound **1** showed similar antibacterial activities against *Bacillus subtilis* to ampicillin as a positive control drug. Our results threw some light on the structure-activity relationships and potential applications of these compounds.

## Supplementary Files

Supplementary File 1 Supplementary Information (PDF, 1890 KB)
