# Supplementary material for: New Spirotetronate Antibiotics, Lobophorins H and I, from a South China Sea-Derived Streptomyces sp. 12A35
Source: Mar Drugs. 2013 Oct 15;11(10):3891–901. doi: 10.3390/md11103891 (PMC3826141; doi:10.3390/md11103891)

Supplementary Information

Figure S1. HRESIMS of lobophorin H (**1**)……………………………………………………...…2
Figure S2. ^{1}H NMR (600 MHz, $CDCl_3$) spectrum of lobophorin H (**1**)…………………………..2
Figure S3. ^{13}C NMR (150 MHz, $CDCl_3$) spectrum of lobophorin H (**1**)…………………………3
Figure S4. HSQC of lobophorin H (**1**)…………………………………………………………..…3
Figure S5. HMBC of lobophorin H (**1**)……………………………………………………………..4
Figure S6. ^{1}H-^{1}H COSY of lobophorin H (**1**)…………………………………………………4
Figure S7. NOESY of lobophorin H (**1**)…………………………………………………………..5
Figure S8. HRESIMS of lobophorin I (**2**)…………………………………………………………5
Figure S9. ^{1}H NMR (600 MHz, $CDCl_3$) spectrum of lobophorin I (**2**)…………………………6
Figure S10. ^{13}C NMR (150 MHz, $CDCl_3$) spectrum of lobophorin I (**2**)………………………6
Figure S11. HSQC of lobophorin I (**2**)……………………………………………………………7
Figure S12. HMBC of lobophorin I (**2**)……………………………………………………………7
Figure S13. ^{1}H-^{1}H COSY of lobophorin I (**2**)………………………………………..…………8
Figure S14. HRESIMS of *O*-β-kijanosyl-(1→17)-kijanolide (**3**)…………………………...….8
Figure S15. ^{1}H NMR (600 MHz, $CDCl_3$) spectrum of *O*-β-kijanosyl-(1→17)-kijanolide (**3**)….9
Figure S16. ^{13}C NMR (150 MHz, $CDCl_3$) spectrum of *O*-β-kijanosyl-(1→17)-kijanolide (**3**)…9
Figure S17. HRESIMS of lobophorin B (**4**)………………………………..………………10
Figure S18. ^{1}H NMR (600 MHz, $CDCl_3$) spectrum of lobophorin B (**4**)…….….…………….10
Figure S19. ^{13}C NMR (150 MHz, $CDCl_3$) spectrum of lobophorin B (**4**)…………………….11
Figure S20. HRESIMS of lobophorin F (**5**)…………………………………………………….11
Figure S21. ^{1}H NMR (600 MHz, $CDCl_3$) spectrum of lobophorin F (**5**)………………………12

Figure S1. HRESIMS of lobophorin H (**1**).

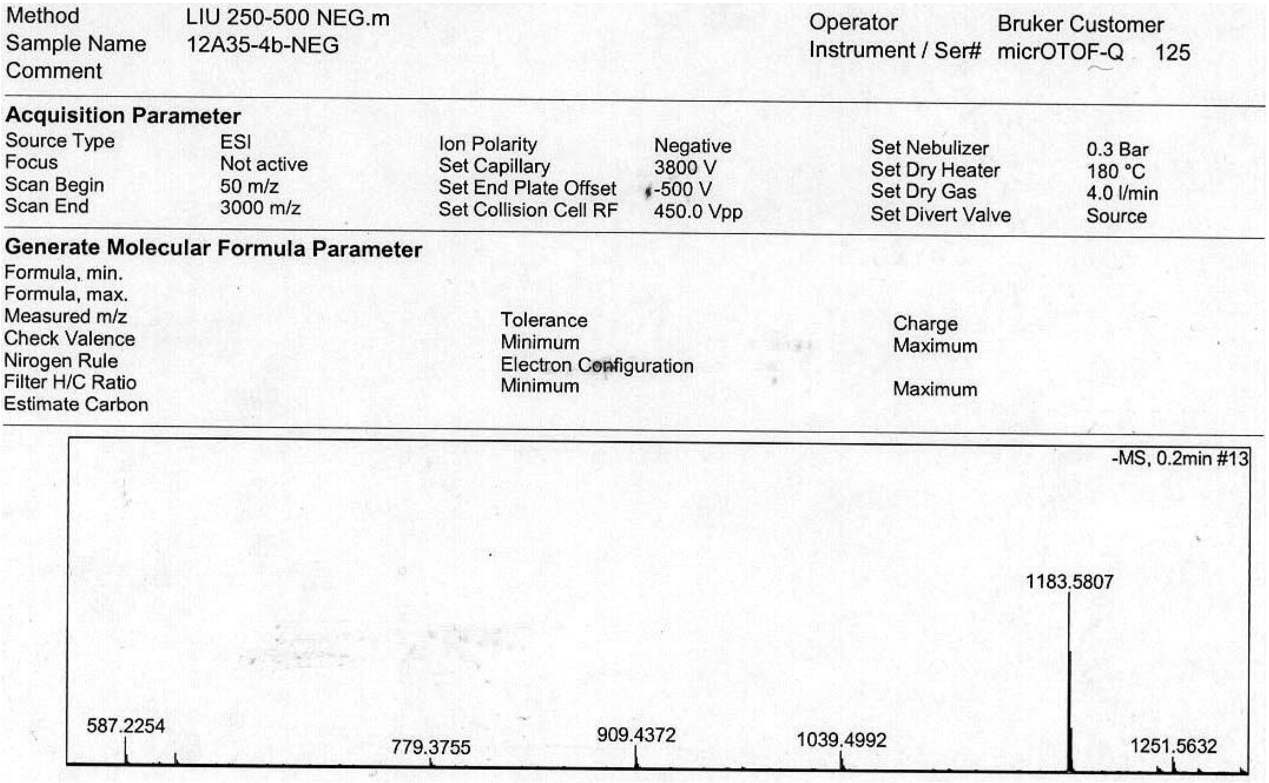

Figure S2. ^{1}H NMR (600 MHz, $CDCl_3$) spectrum of lobophorin H (**1**).

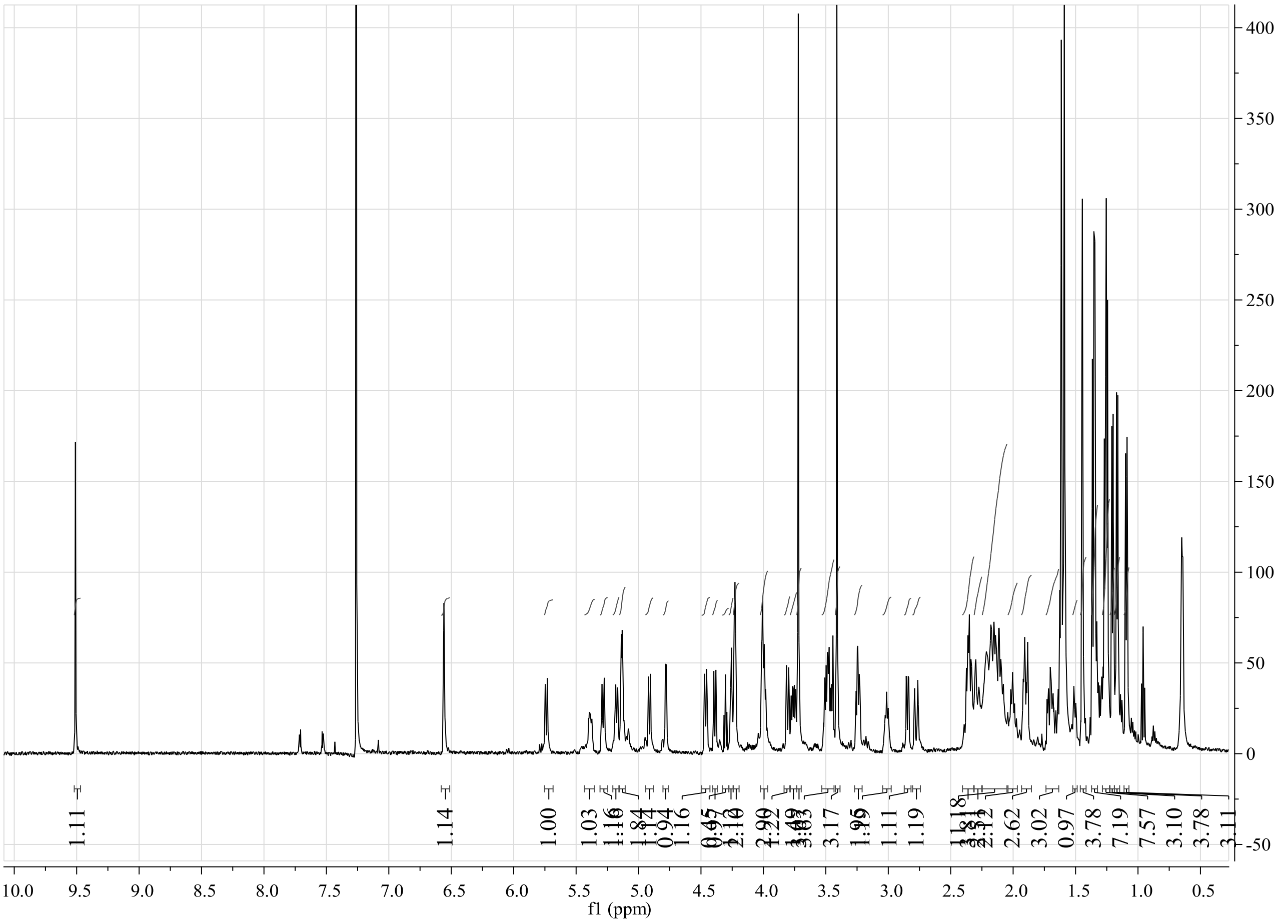

Figure S3. ^{13}C NMR (150 MHz, $CDCl_3$) spectrum of lobophorin H (**1**).

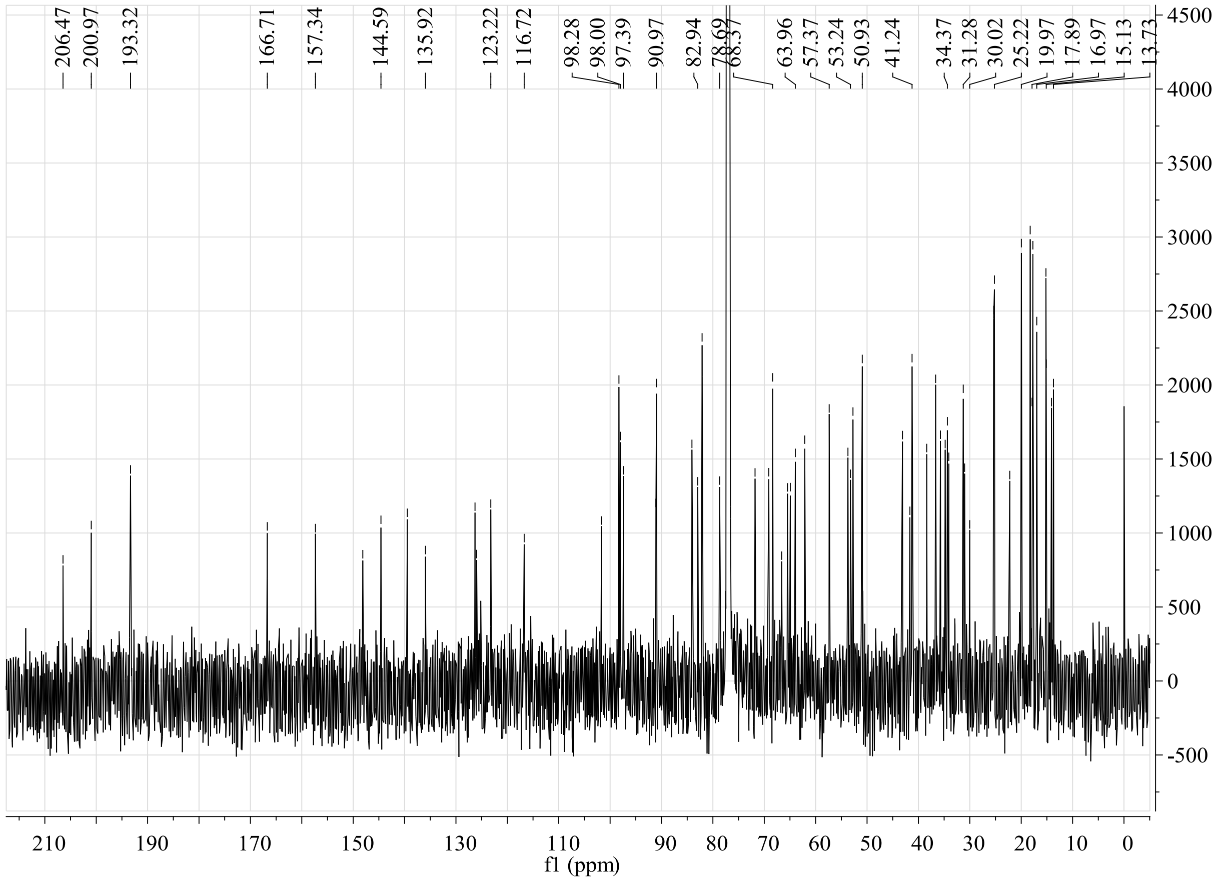

Figure S4. HSQC of lobophorin H (**1**).

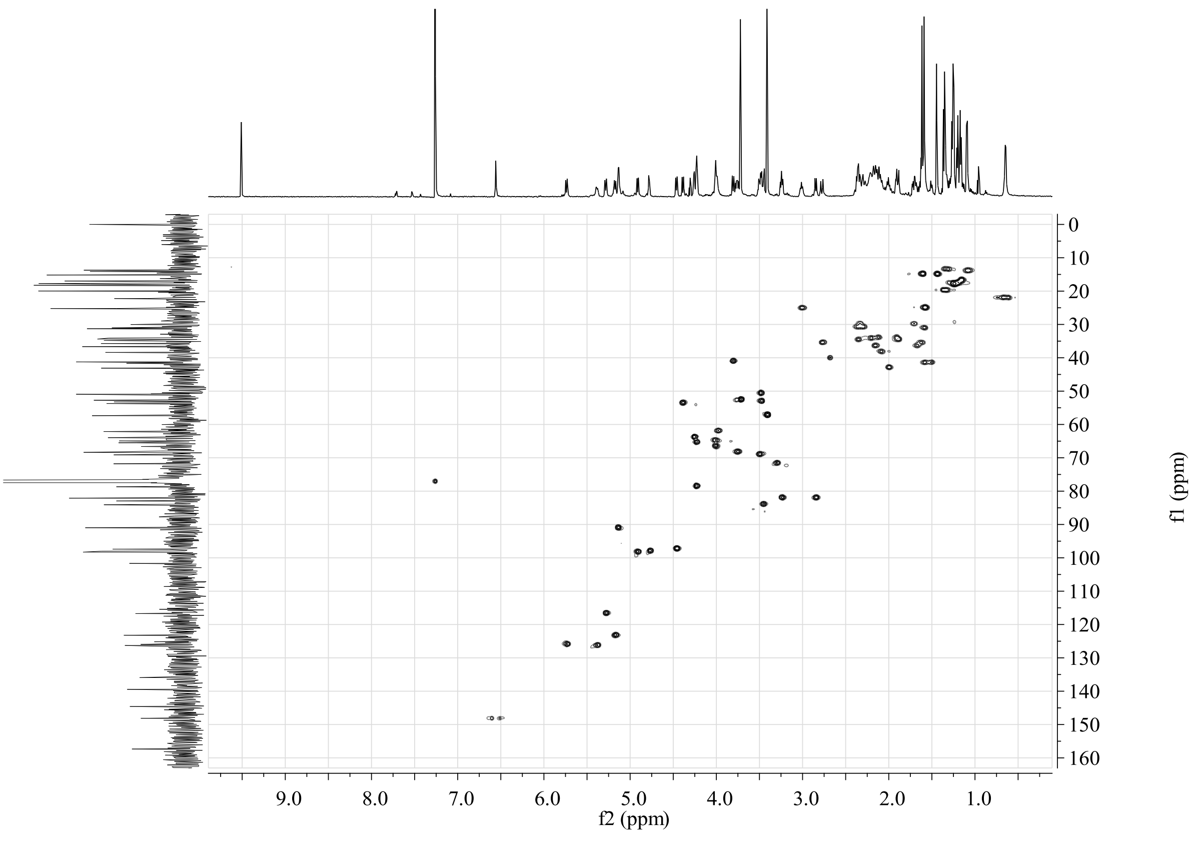

Figure S5. HMBC of lobophorin H (**1**).

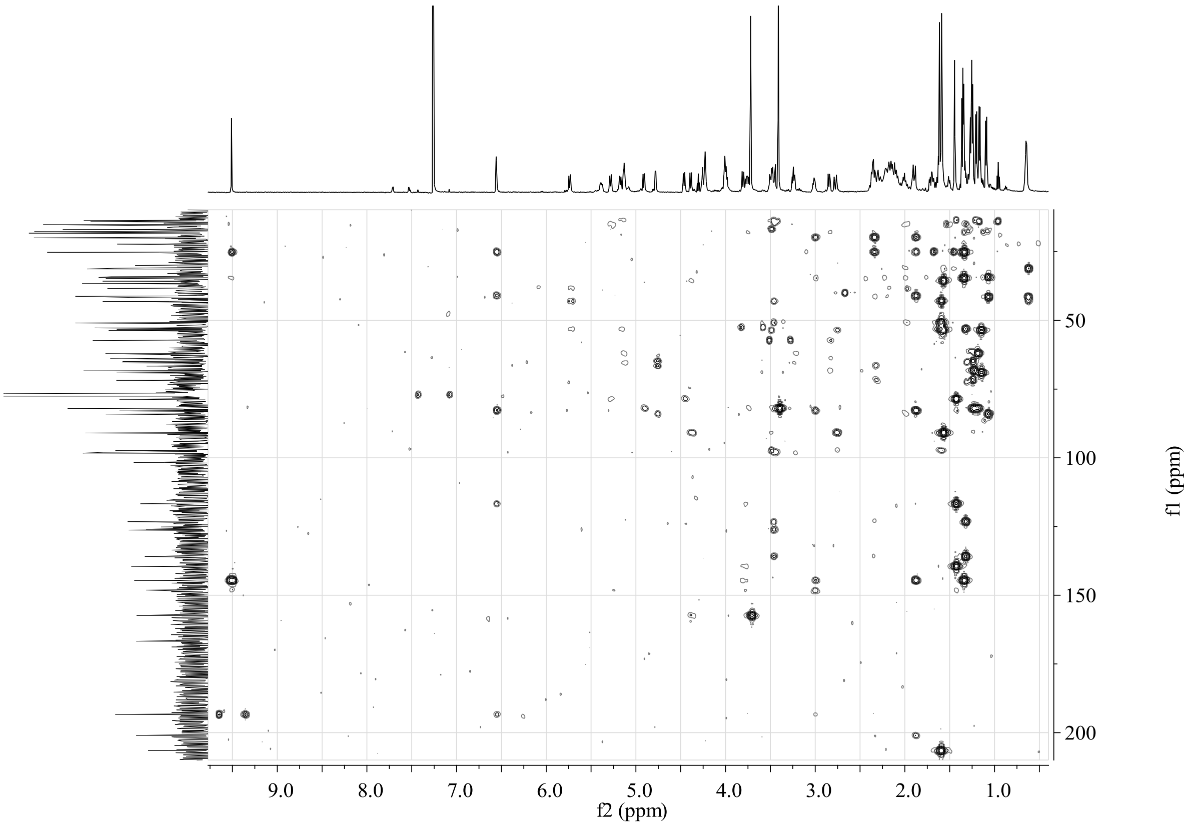

Figure S6. ¹H-¹H COSY of lobophorin H (**1**).

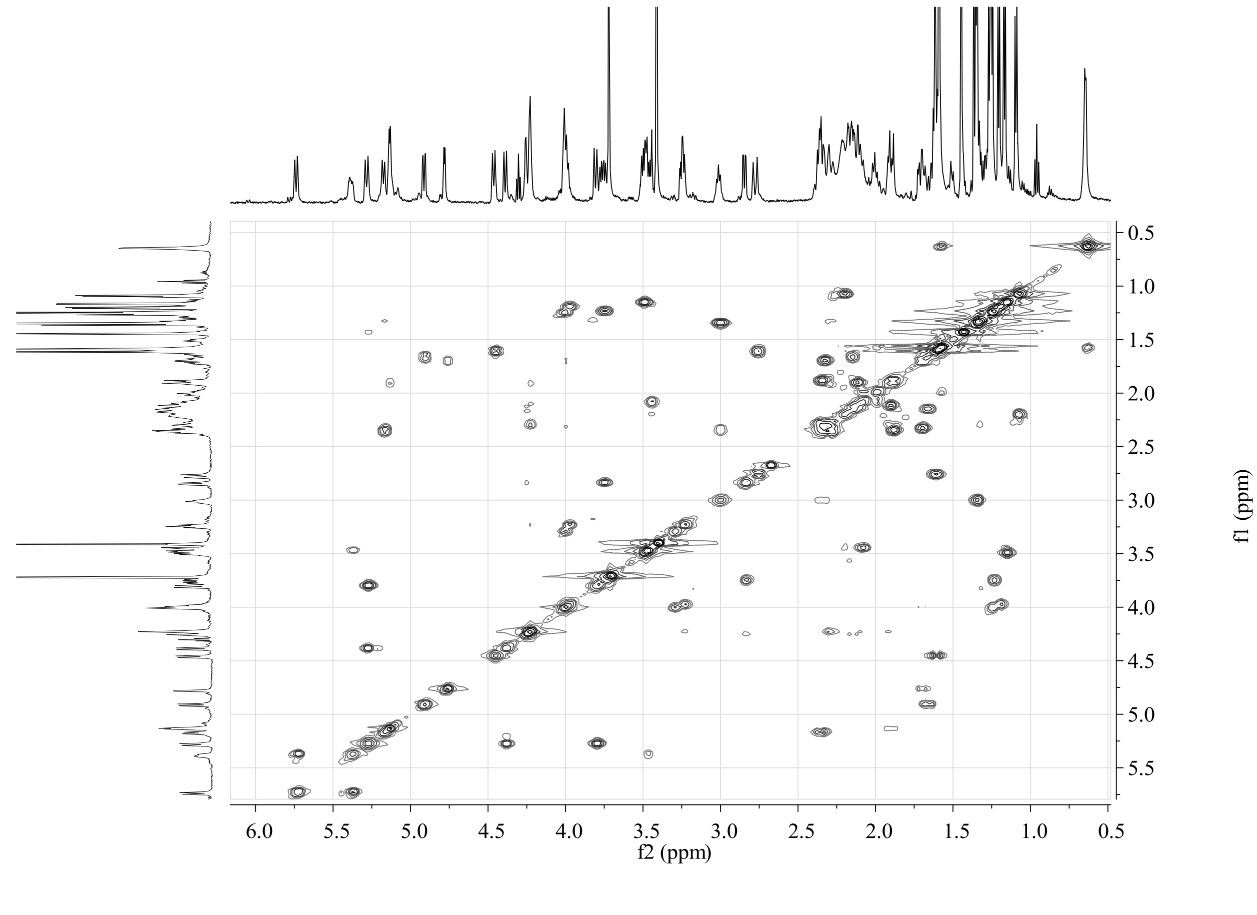

Figure S7. NOESY of lobophorin H (**1**).

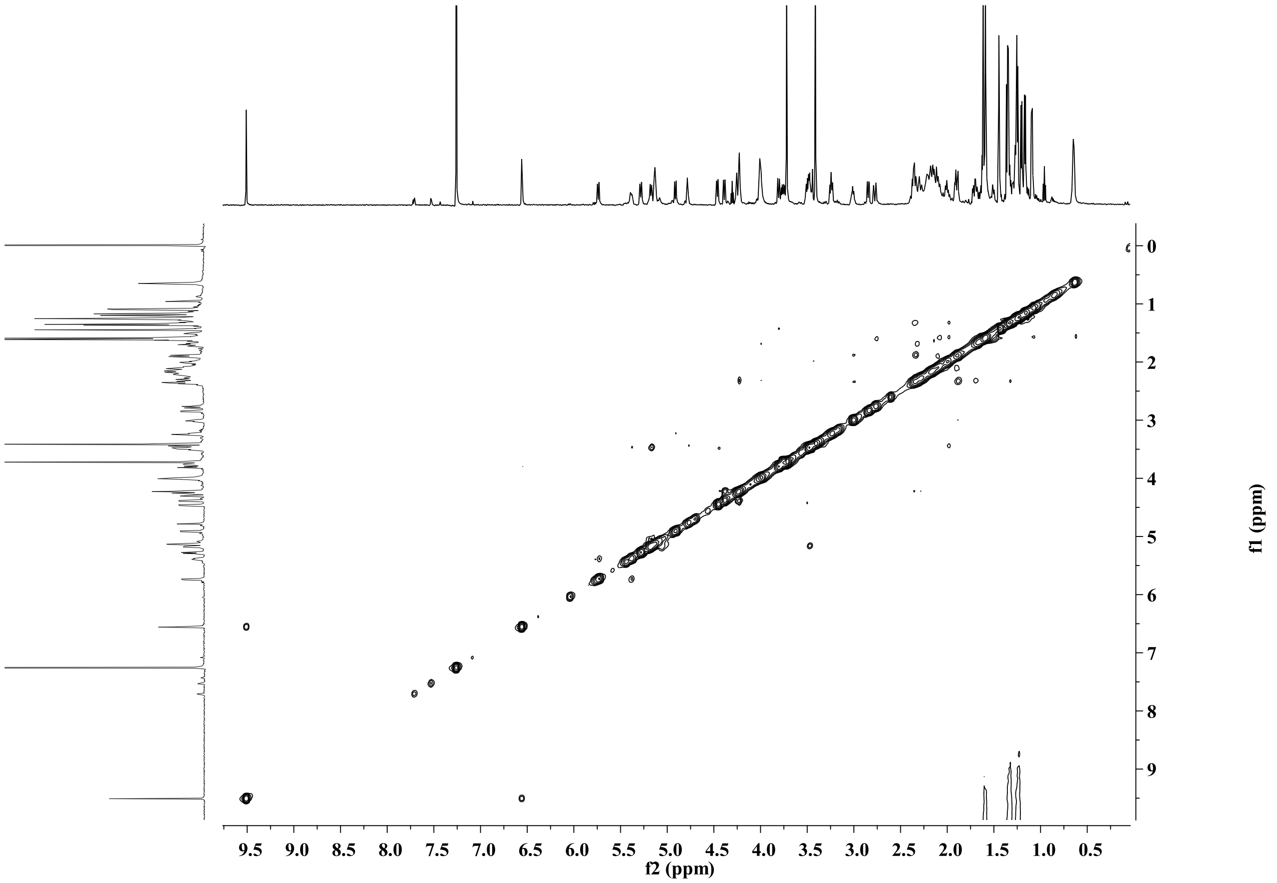

Figure S8. HRESIMS of lobophorin I (**2**).

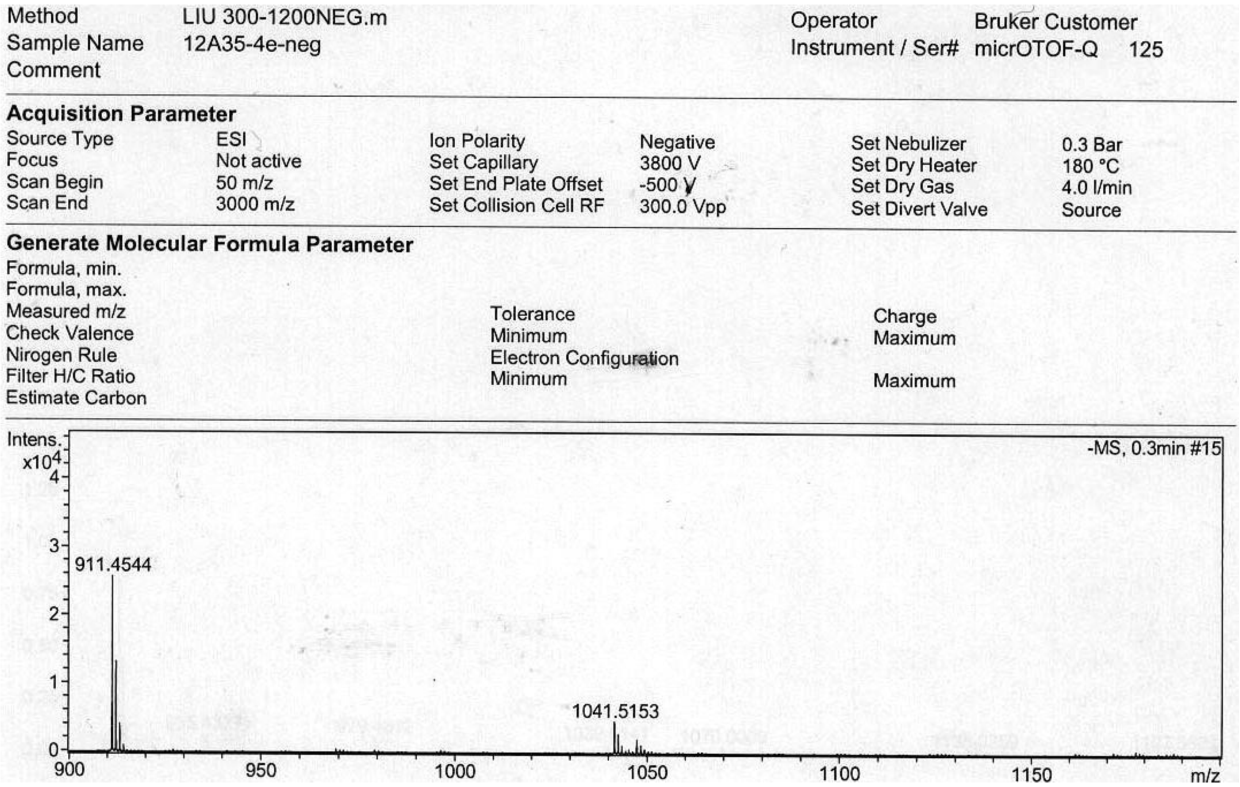

Figure S9. ^{1}H NMR (600 MHz, $CDCl_3$) spectrum of lobophorin I (**2**).

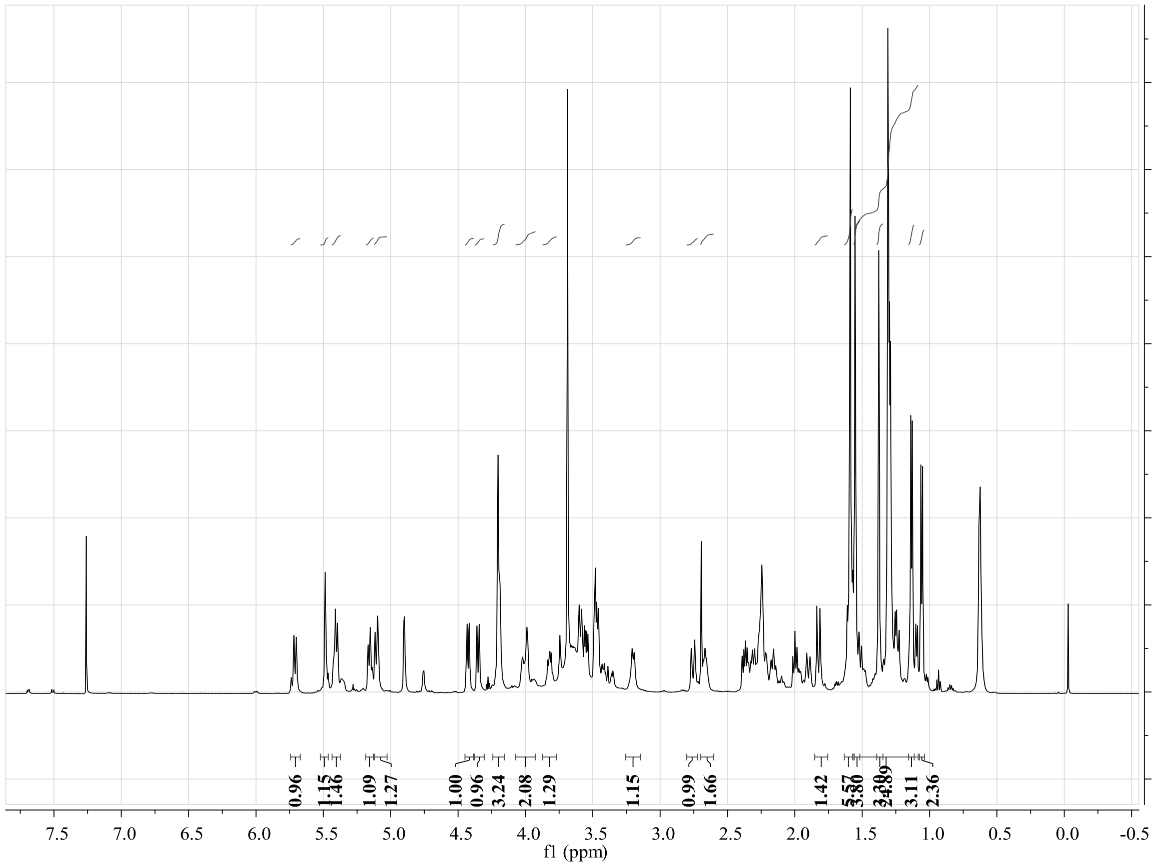

Figure S10. ^{13}C NMR (150 MHz, $CDCl_3$) spectrum of lobophorin I (**2**).

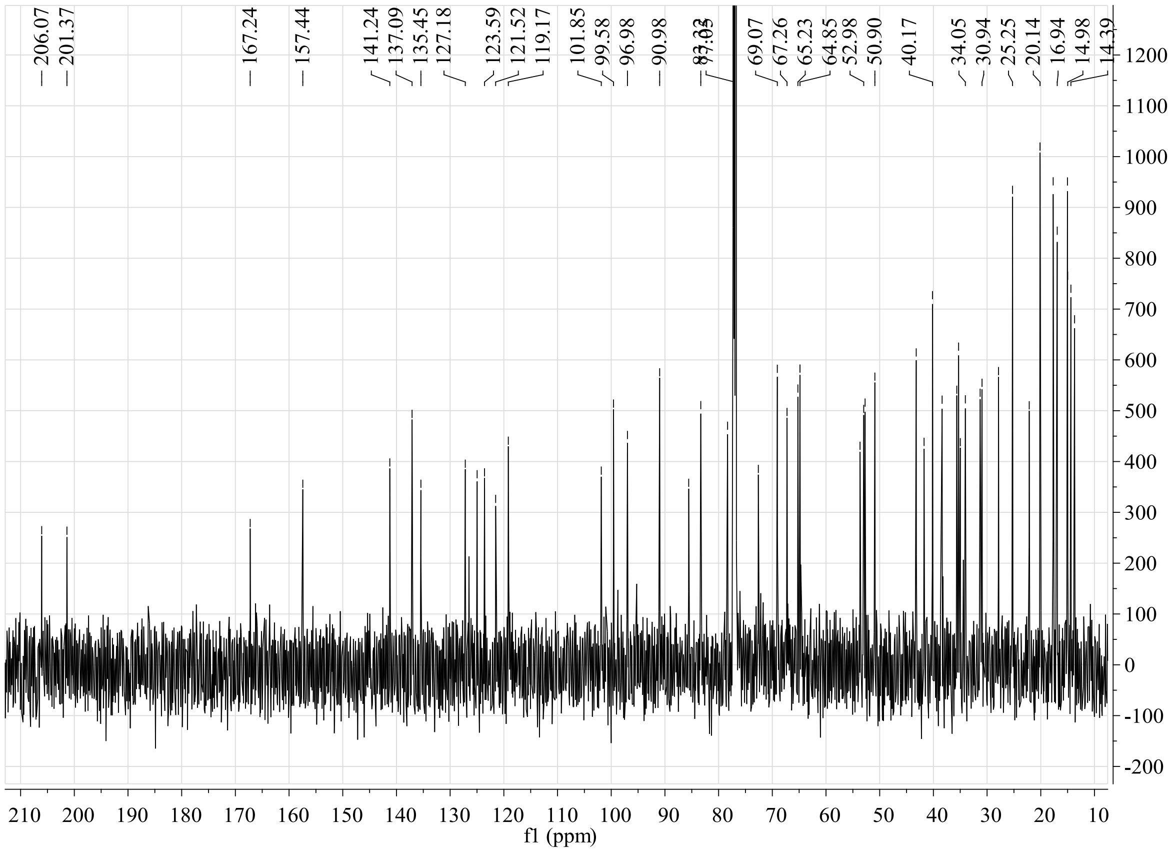

Figure S11. HSQC of lobophorin I (**2**).

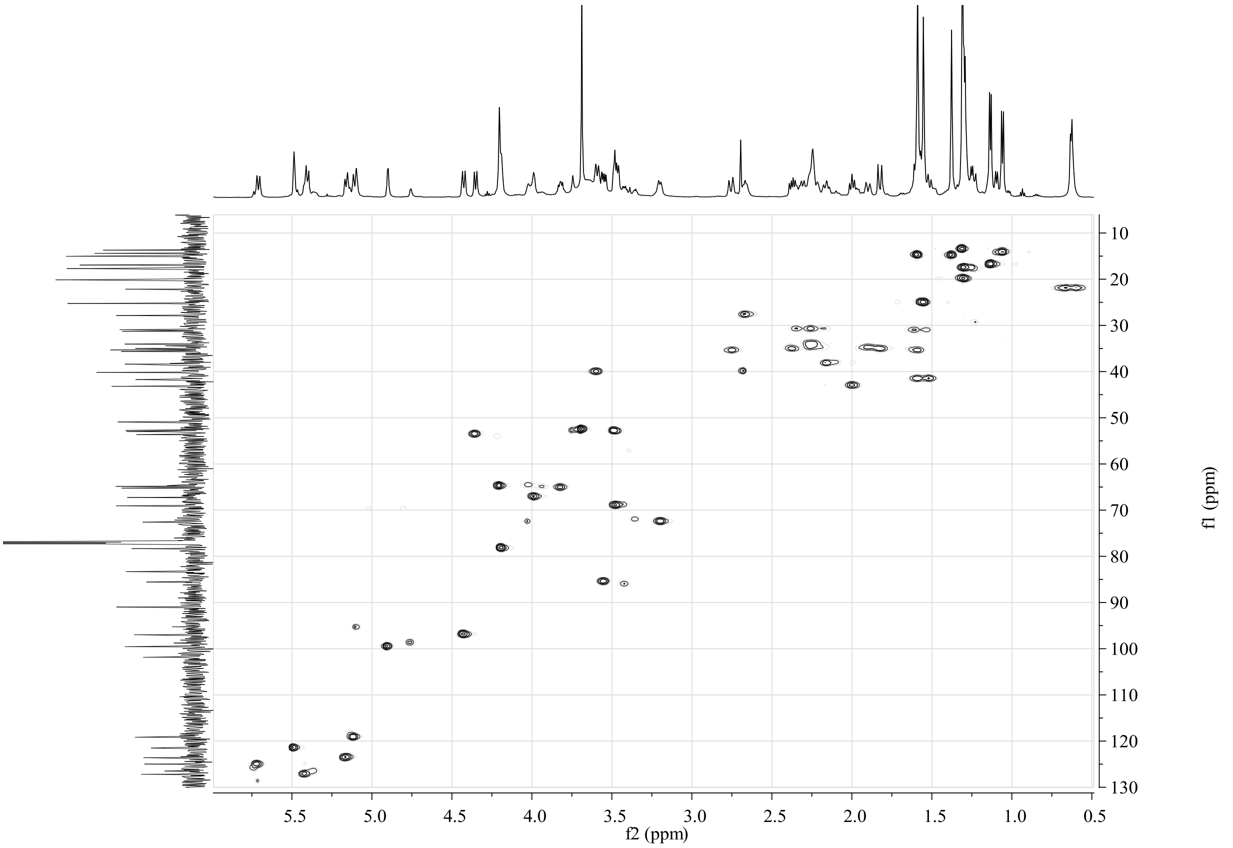

Figure S12. HMBC of lobophorin I (**2**).

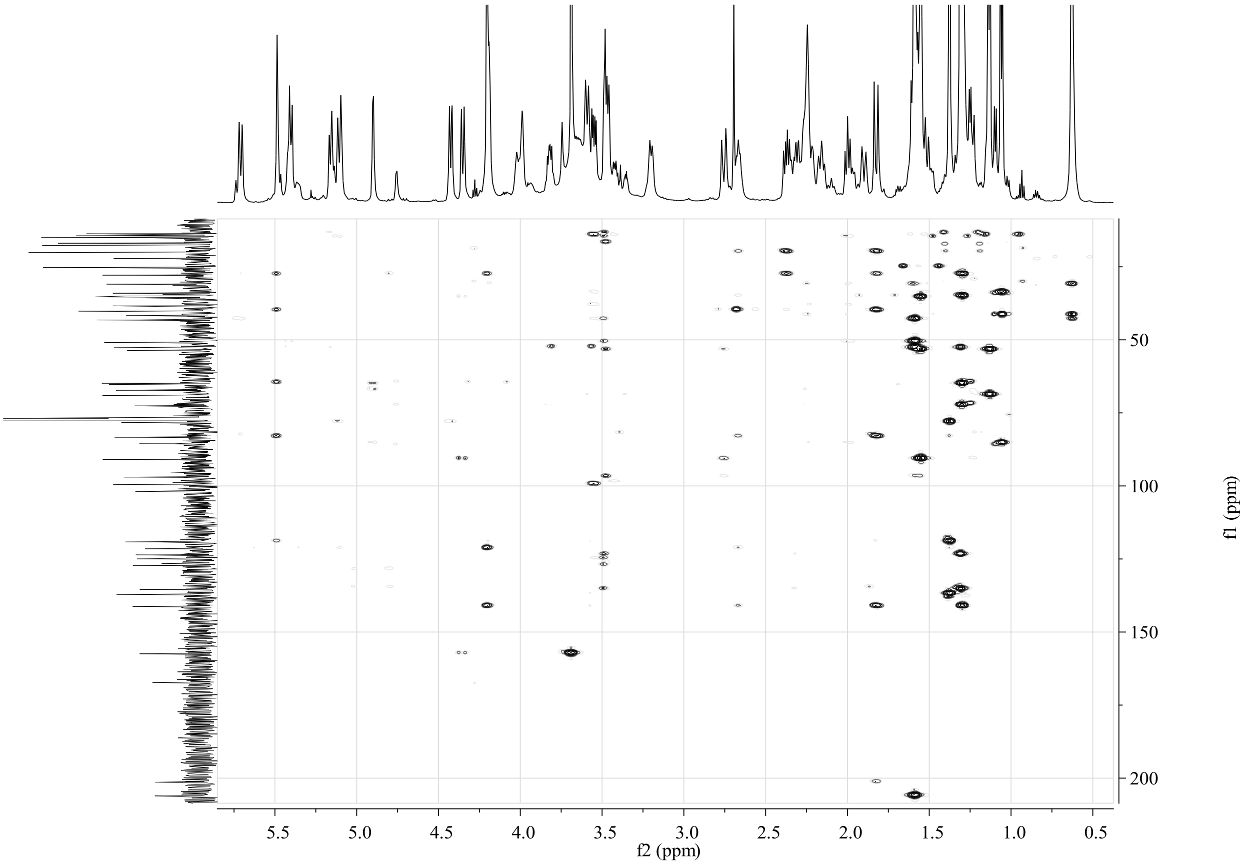

Figure S13. ^{1}H-^{1}H COSY of lobophorin I (**2**).

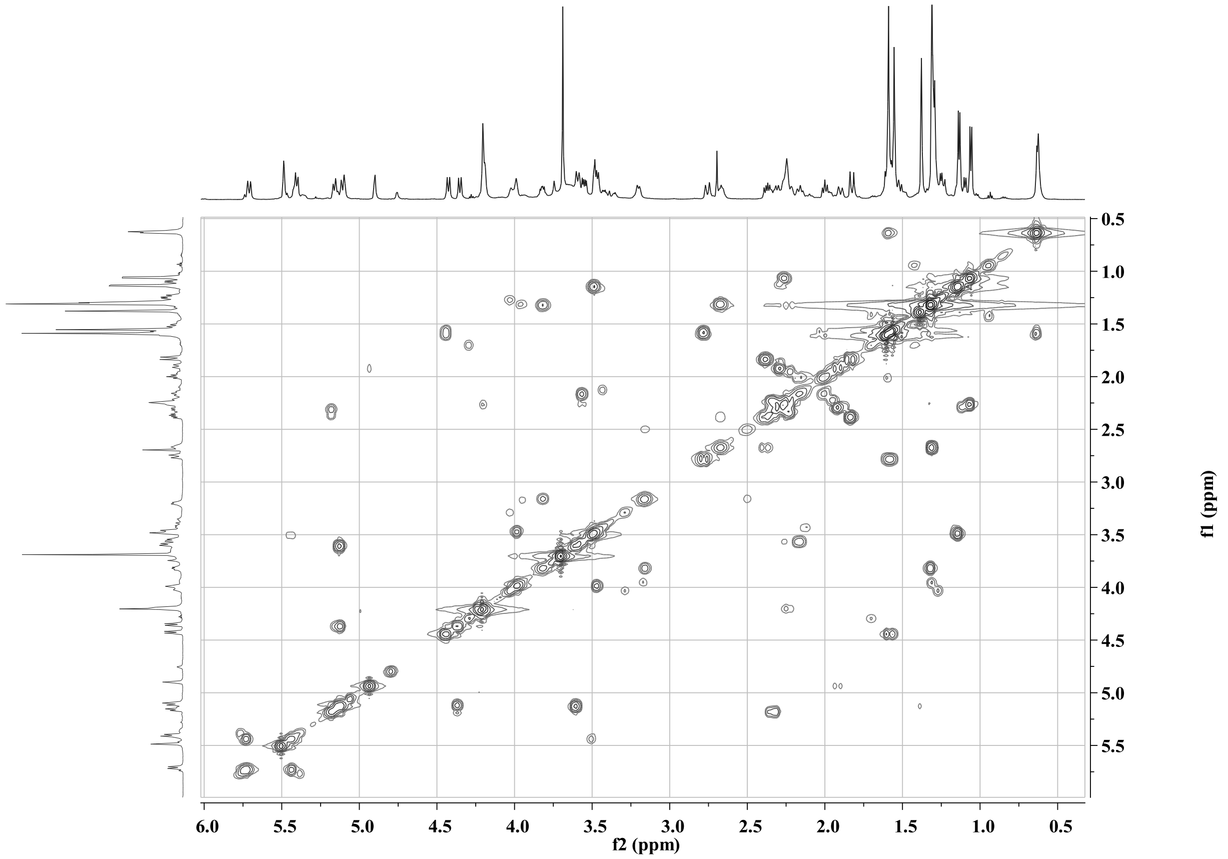

Figure S14. HRESIMS of *O*-β-kijanosyl-(1→17)-kijanolide (**3**).

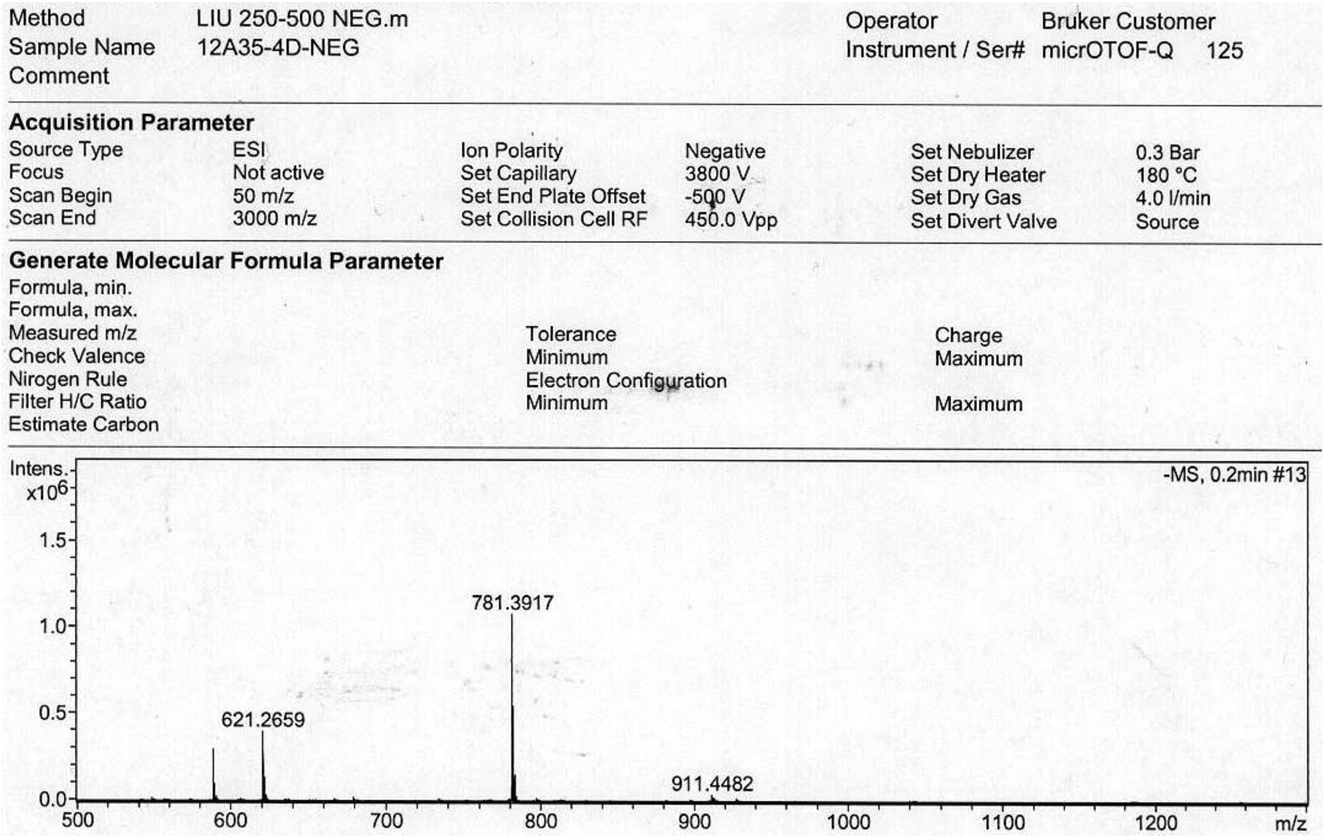

Figure S15. ^{1}H NMR (600 MHz, $CDCl_3$) spectrum of *O*-β-kijanosyl-(1→17)-kijanolide (**3**).

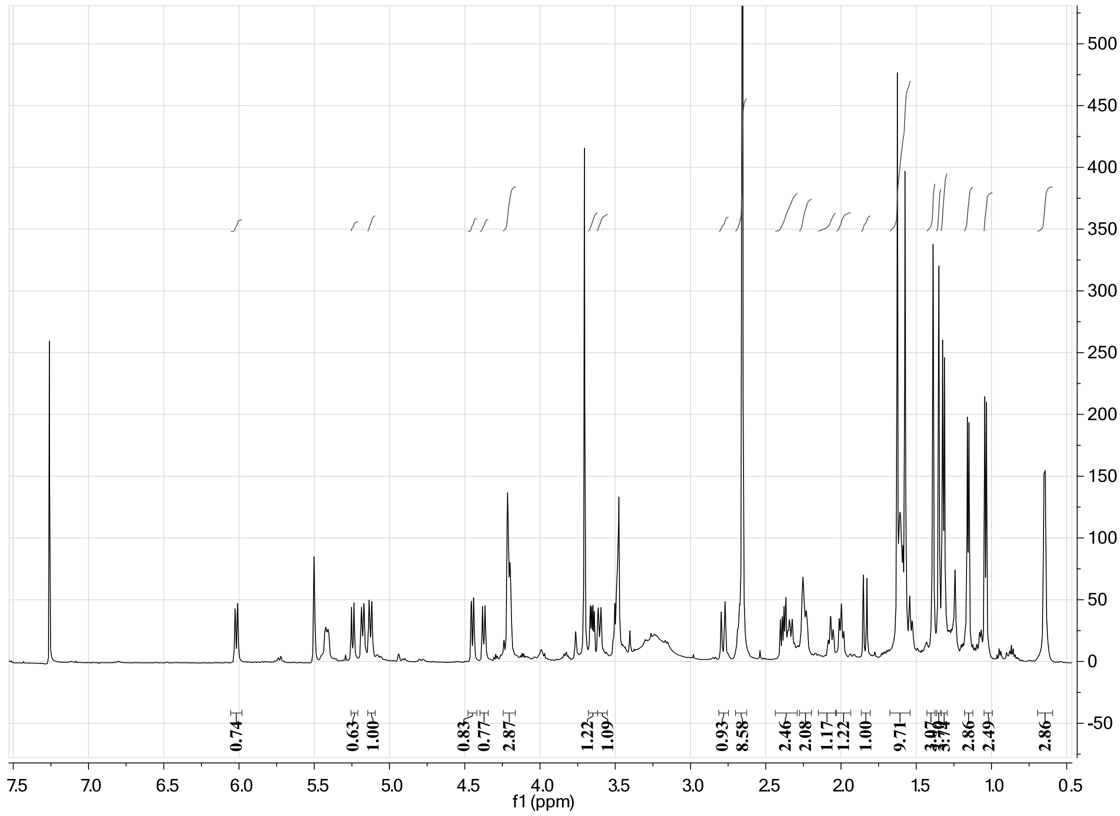

Figure S16. ^{13}C NMR (150 MHz, $CDCl_3$) spectrum of *O*-β-kijanosyl-(1→17)-kijanolide (**3**).

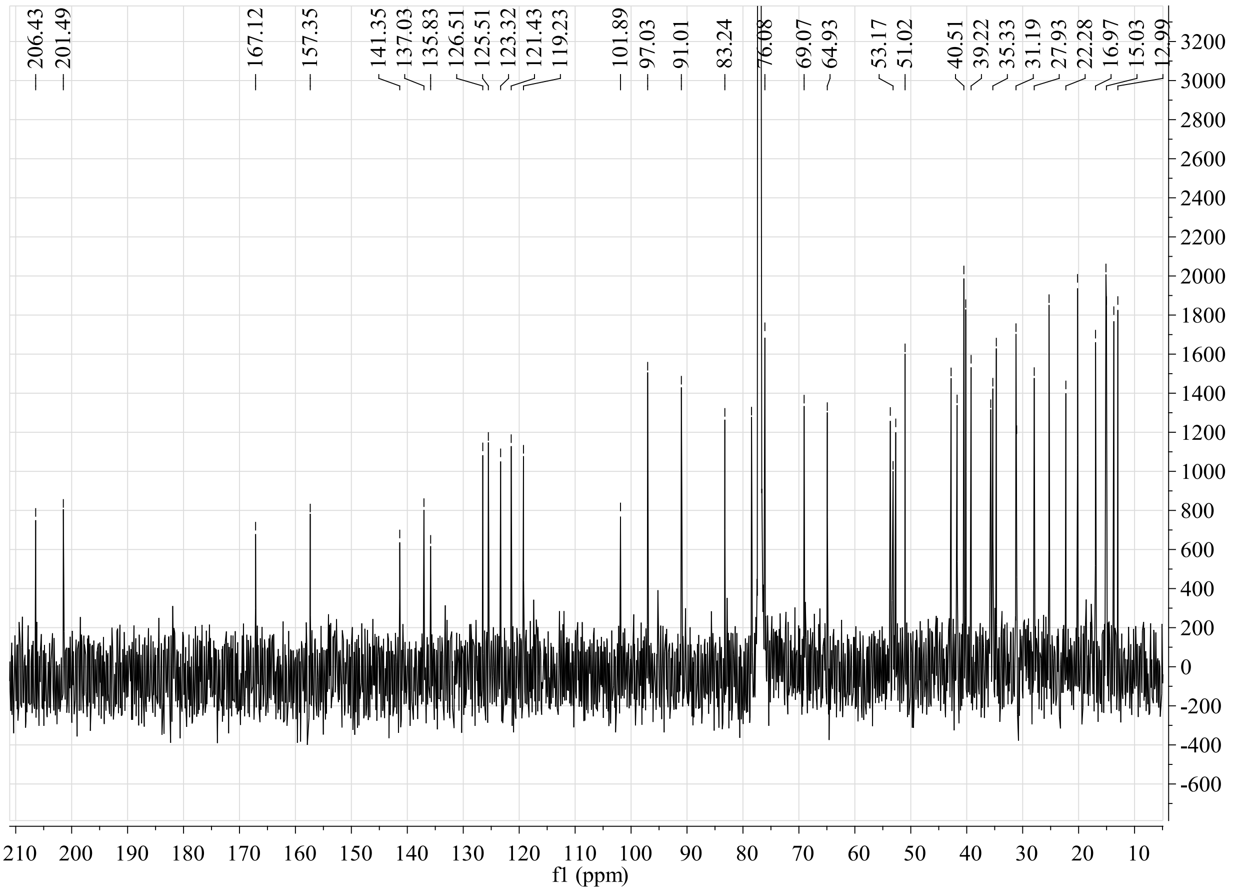

Figure S17. HRESIMS of lobophorin B (**4**).

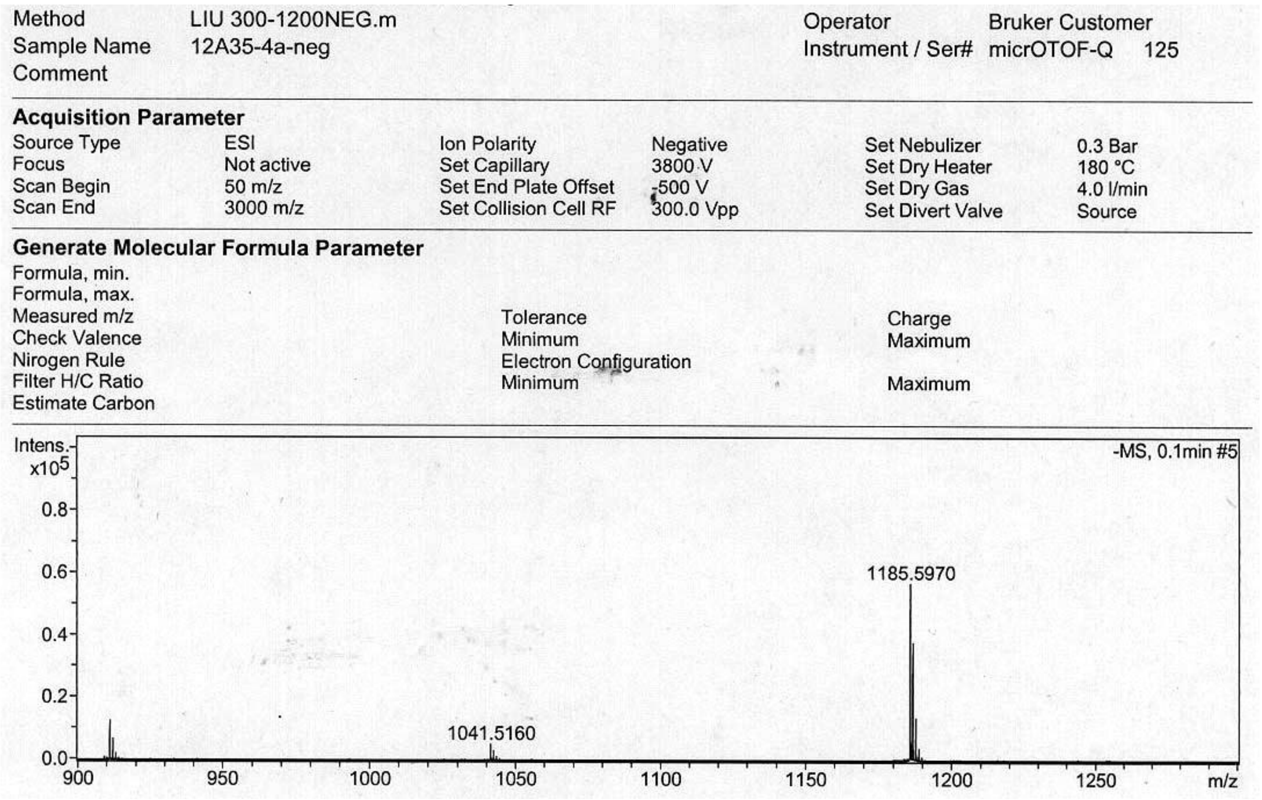

Figure S18. ^{1}H NMR (600 MHz, $CDCl_3$) spectrum of lobophorin B (**4**).

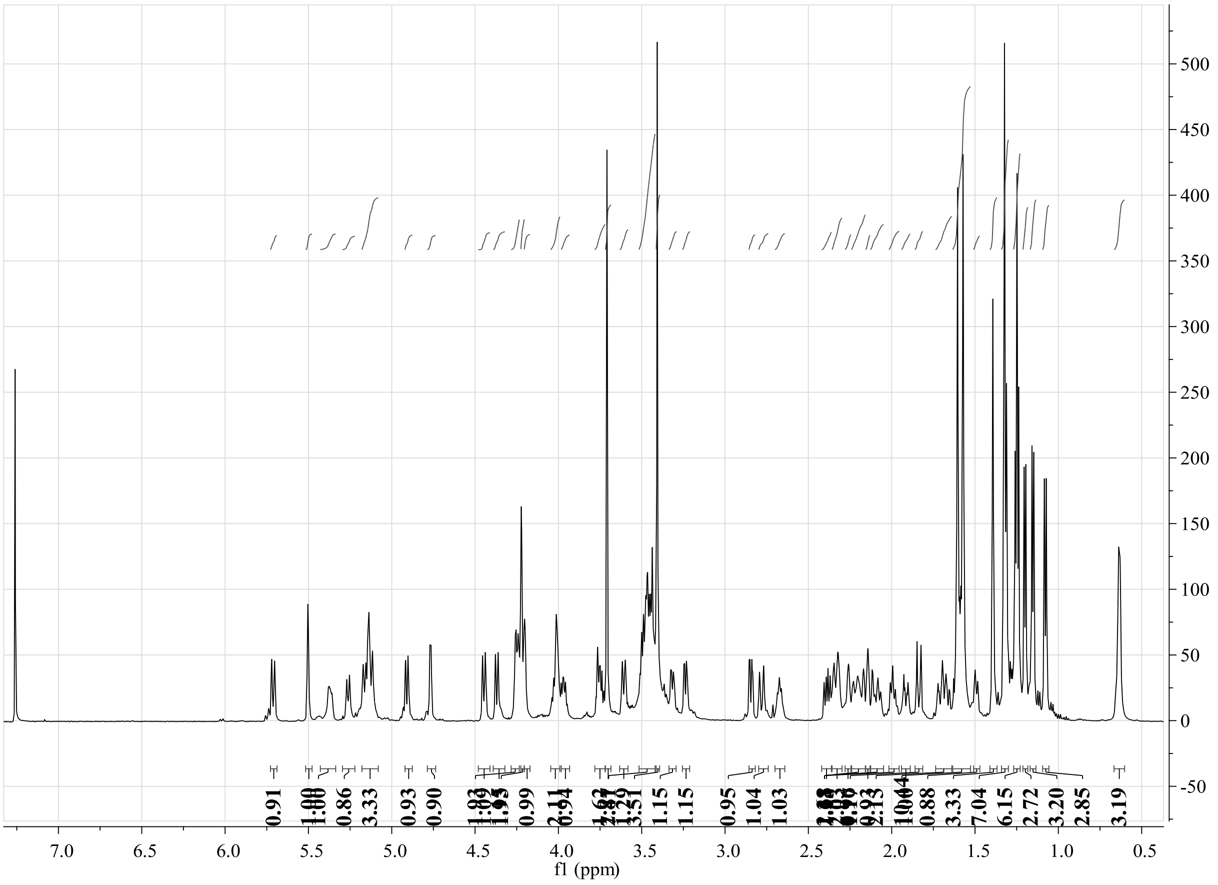

Figure S19. ^{13}C NMR (150 MHz, $CDCl_3$) spectrum of lobophorin B (**4**).

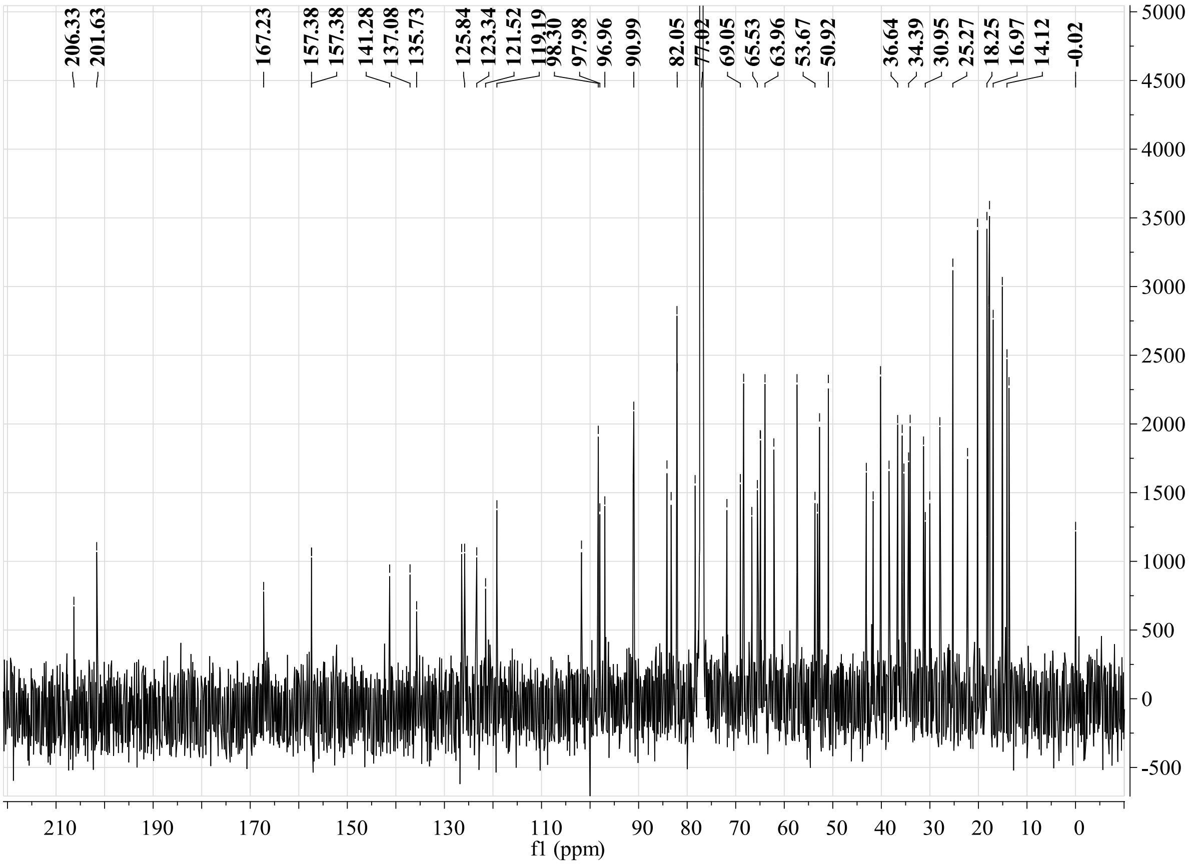

Figure S20. HRESIMS of lobophorin F (**5**).

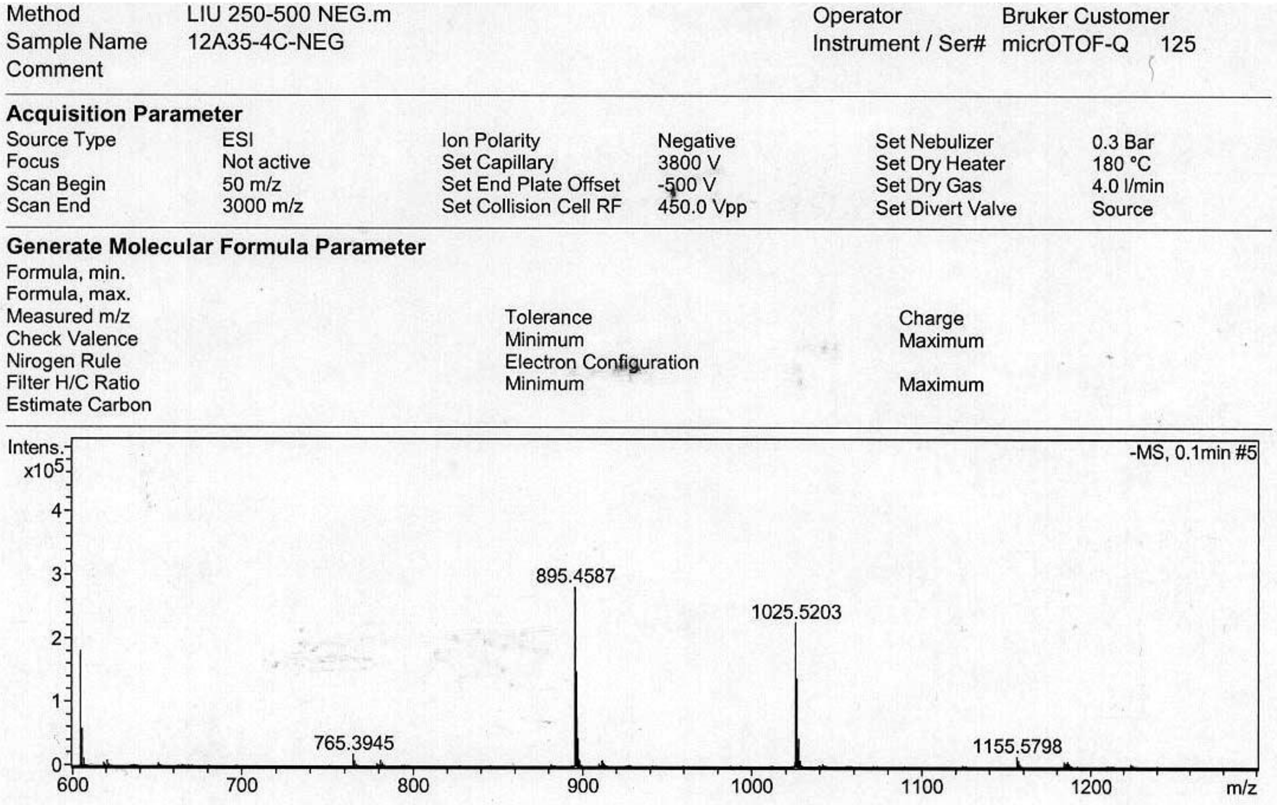

Figure S21. ^{1}H NMR (600 MHz, $CDCl_3$) spectrum of lobophorin F (**5**).

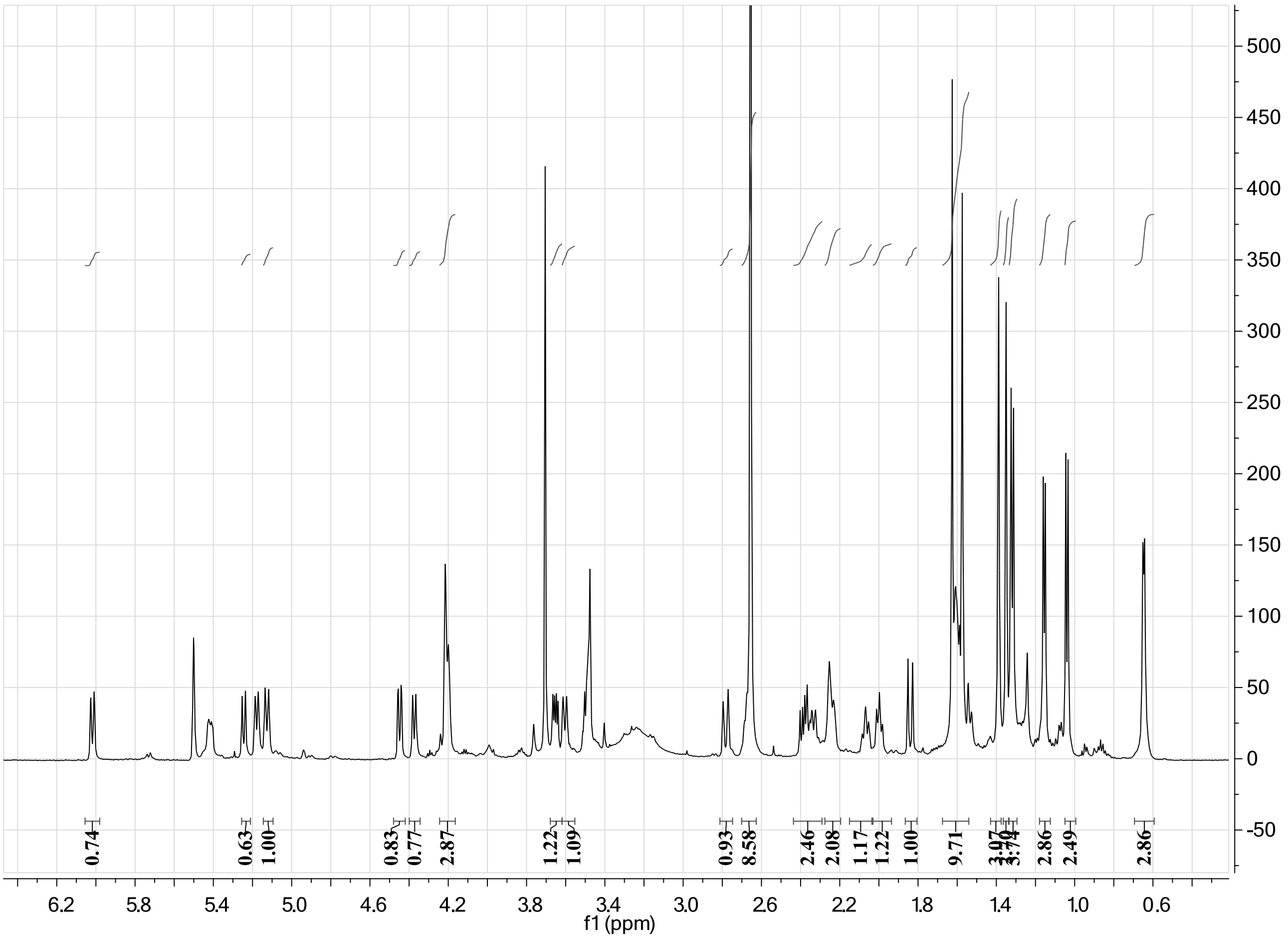

Supplement: Supplementary File 1 — Supplementary Information (PDF, 1890 KB) [file marinedrugs-11-03891-s001.pdf]
